# Up-Regulated Dicer Expression in Patients with Cutaneous Melanoma

**DOI:** 10.1371/journal.pone.0020494

**Published:** 2011-06-17

**Authors:** Zhihai Ma, Helen Swede, David Cassarino, Elizabeth Fleming, Andrew Fire, Soheil S. Dadras

**Affiliations:** 1 Department of Genetics, Stanford University School of Medicine, Stanford, California, United States of America; 2 Department of Community Medicine and Health Care, University of Connecticut Health Center, Farmington, Connecticut, United States of America; 3 Department of Pathology, Southern California Permanente Medical Group, Los Angeles, California, United States of America; 4 Department of Genetics/Developmental Biology, University of Connecticut Health Center, Farmington, Connecticut, United States of America; 5 Department of Dermatology, University of Connecticut Health Center, Farmington, Connecticut, United States of America; University of Hong Kong, Hong Kong

## Abstract

**Background:**

MicroRNAs (miRNAs) are small non-coding RNAs (18–24 nucleotides) that have recently been shown to regulate gene expression during cancer progression. Dicer, a central enzyme in the multi-component miRNA biogenesis pathway, is involved in cutting precursor miRNAs to functionally mature forms. Emerging evidence shows that Dicer expression is deregulated in some human malignancies and it correlates with tumor progression, yet this role has not yet been investigated in skin cancers.

**Methods and Findings:**

Using an anti-human monoclonal antibody against Dicer and immunohistochemistry, we compared the expression of Dicer protein among 404 clinically annotated controls and skin tumors consisting of melanocytic nevi (n = 71), a variety of melanomas (n = 223), carcinomas (n = 73) and sarcomas (n = 12). Results showed a cell-specific up-regulated Dicer in 81% of cutaneous, 80% of acrolentiginous and 96% of metastatic melanoma specimens compared to carcinoma or sarcoma specimens (*P*<0.0001). The expression of Dicer was significantly higher in melanomas compared to benign melanocytic nevi (*P*<0.0001). In patients with cutaneous melanomas, Dicer up-regulation was found to be significantly associated with an increased tumor mitotic index (*P* = 0.04), Breslow's depth of invasion (*P* = 0.03), nodal metastasis (*P* = 0.04) and a higher American Joint Committee on Caner (AJCC) clinical stage (*P* = 0.009). Using western blot analysis, we confirmed the cell-specific up-regulation of Dicer protein *in vitro*. A pooled-analysis on mRNA profiling in cutaneous tumors showed up-regulation of Dicer at the RNA level in cutaneous melanoma, also showing deregulation of other enzymes that participate in the biogenesis and maturation of canonical miRNAs.

**Conclusions:**

Increased Dicer expression may be a clinically useful biomarker for patients with cutaneous melanoma. Understanding deregulation of Dicer and its influence on miRNA maturation is needed to predict the susceptibility of melanoma patients to miRNA-based therapy in the future.

## Introduction

Small (18–24 nucleotides) non-coding RNAs, including microRNAs (miRNAs), regulate gene expression in many biological processes [1] and in human cancers [2]. miRNAs down-regulate expression of specific target genes, in part drawing specificity from seed sequence pairing within the 3′-untranslated region of target mRNAs leading to translational repression and/or mRNA degradation [3]. Specific miRNAs can function as tumor suppressor genes or oncogenes (oncomirs) [4] where deregulated miRNA expression has been demonstrated in a variety of human cancers including chronic lymphocytic leukemia [5], lung cancer [6], colorectal neoplasia [7] and pancreatic endocrine and acinar tumors [8]. Global miRNA expression profiling has provided some evidence that the expression of certain miRNAs is deregulated during melanoma progression. While these studies are limited to cell lines [9], [10], [11] and metastatic lesions [12], little is known about the role of miRNAs or their biogenesis pathway in clinical primary melanoma [13], [14].

Dicer, a member of the RNase III family of double-stranded RNases, is a central enzyme in a multi-component miRNA biogenesis pathway where the Drosha/DGCR8 complex and Dicer act sequentially to crop long primary and precursor miRNAs into functionally mature miRNAs [15]. The expression levels of the processing components themselves are certainly candidates for deregulation in the development and the progression of human cancers. For example, high dicer expression is a poor prognostic factor in patients with prostate adenocarcinoma [16], whereas low Dicer expression is a poor prognostic factor in lung [17] and ovarian [18] carcinoma.

Although changes in the expression level of Dicer, and possibly other miRNA processing enzymes, are of clinical significance in some cancers, these alterations or their clinical consequences in melanoma or other skin cancers remain unknown.

We demonstrate herein that Dicer protein is specifically up-regulated in melanoma compared to other skin cancers such as carcinomas and sarcomas. This up-regulation is further specific to melanoma subtype and is significantly associated with clinical stage in patients with cutaneous melanoma. Additional analyses of Dicer levels in tissue culture cells support a general up-regulation of Dicer in melanoma and suggest an autonomous up-regulation in the absence of supporting cells.

## Materials and Methods

### Clinical Profile of Tissue Microarrays and Cases

We used seven different tissue microarray (TMA) slides, prepared from formalin-fixed paraffin embedded (FFPE) specimens, representing a wide variety of human skin tumors, both benign and malignant, from 404 different patients arrayed onto slides at 80 to 100 cores per slide (in duplicates or triplicates). This set also included complete tumor FFPE sections. The TMA slides included normal tissue (skin and other organs), melanocytic nevi (compound, intradermal and blue), primary melanomas (acrolentiginous, cutaneous, desmoplastic, mucosal, ocular), metastatic melanomas, squamous cell carcinoma, adenocarcinoma (eccrine, sebaceous and metastatic), basal cell carcinoma, sarcomas (dermatofibrosarcoma protuberans and fibrosarcomas), neurofibromas, and benign and malignant schwannomas. We purchased TMA slides ME1001, ME801, SK803 and BC21011 from US Biomax, Inc. (Rockville, MD) and CS38-01-001 from Cybrdi (Rockville, MD). The squamous or melanocytic differentiation of tumors was confirmed by immunohistochemical staining for cytokeratins or HMB-45, respectively, by the manufacturers. DC-1 and DC-2 TMAs were built at Stanford University Pathology Department (DC). For most TMAs, the information on age, sex and anatomic sites were available. For primary melanomas arrayed on DC-1 and DC-2 TMAs, the information on age, tumor thickness (Breslow's depth, Clark's level of invasion and histological type were available. Complete tumor sections were also examined that included cutaneous melanomas (n = 19), metastatic melanomas to lymph node (n = 5) and common nevi (n = 9). At least two pathologists/dermatopathologists (SSD and/or DC) confirmed all diagnoses. The institutional review boards of the Stanford University Medical Center and the University of Connecticut Health Center approved this protocol.

### Patients

Out of 95 patients diagnosed with primary cutaneous melanoma, we had complete clinical follow-up information for 19 patients (mean = 26.6 month, range 7 to 64 months). The majority of cases were obtained from the Stanford University Pathology archive from 1997-2006. Clinical information included gender, age, anatomic site of the primary tumor, relapse-free survival, American Joint Committee on Cancer (AJCC) pathological (tumor, node, metastasis) stage [19], site of first metastasis, sentinel (SLN) and non-sentinel lymph node (NSLN), distant metastasis and overall survival. Distant metastasis was defined as either distant nodal or visceral. Histological information included tumor thickness (mm), Clark's level of invasion, ulceration, histological subtype, regression, and mitotic index (mitoses per square millimeter) as previously described [20]. At least two pathologists confirmed all the diagnoses of primary and metastatic melanomas. The institutional review board of the Stanford University Medical Center approved this protocol.

### Immunohistochemistry and Statistical Analysis

Immunostaining for all cases (TMAs and complete sections) was performed on 4-mm-thick FFPE sections mounted on charged slides and incubated at 60°C overnight. All slides were incubated with the anti-human Dicer monoclonal antibody through Clonegene (1∶100, clone mab 13D6, Hartford, CT), generously provided by Dr. Henry Furneaux. We performed antigen retrieval using DakoCytomation Target Retrieval Solution (High pH, Catalogue No. S-3308) and biocare digital decloaking chamber (Biocare Medical, Concord, CA) at 100°C for 10 minutes, followed by treatment of 3% H_2_O_2_ to block the endogenous peroxidase activity. The slides were incubated at room temperature for 1 hour with anti-Dicer antibodies at 1∶100 dilution. Hematoxylin was used for counterstaining. Following the manufacturer's instructions, we developed the immunohistochemical stain using EnVision™+ kit (DAKO, Carpinteria, CA).

The expression of Dicer was examined in normal skin and other organs; Dicer immunoreactivity was seen in the cytoplasm without nuclear staining. A semi-quantitative, four-point ordinal immunoreactivity score was established: “0” reflected the lack of Dicer immunoreactivity and was the most common pattern in normal skin, carcinomas and sarcomas. Weakly positive (“1”) staining was observed in epidermal keratinocytes and melanocytic nevi. Moderately positive (“2”) staining was assigned to modest granular staining. Strongly positive (“3”) staining consisted of diffuse and homogenous staining. Basal levels of Dicer were detected in epidermal keratinocytes. Only staining of tumor cells was scored in comparison to adjacent keratinocytes (internal positive control). Melanophages, specialized macrophages containing dark, brown coarse melanin pigment were not scored. Two investigators scored the stained slides independently. Scores for multiple cores from one case were averaged, and final Dicer scores were categorized into a three-level grouping of Negative, Low (>0 and ≤1.5) or High (>1.6) for analyses that included all 404 patients. For the exploratory analyses (n = 19), a dichotomous breakdown of Negative or Positive (>0) Dicer expression was used. These analyses included the following clinical and histopathological variables: AJCC Stage (I, II, III, IV); Distant Metastases, Non-Sentinel Node Metastases, Organ Metastases, Evidence of Regression, and Ulceration, which were treated as dichotomous variables; Melanoma Histology (Superficial Spreading, Nodular, Acrolentiginous or Lentigo Maligna); Vital status (alive, dead); and, Mitotic Index (mm^2^), Clark's Level and Breslow's depth of invasion, i.e. Tumor Thickness (mm) were treated as continuous variables. Pearson Chi-Square tests were employed to assess categorical levels of Dicer status. Kruskal-Wallis (k = 3) or Mann-Whitney (k = 2) non-parametric tests were used for analyses when Dicer expression was treated as a continuous value as well as for analyses of Mitotic Index, Clarks Level and Breslow's depth of invasion. SPSS version 18.0 was used, and tests were two-sided in all analyses.

### Bioinformatic Pooled Analysis on Gene Expression Profiling

Using NextBio (nextbio.com), we mined and pooled publically available gene expression profiling data interrogating the mRNA levels of the genes encoding all of the known enzymes involved in the canonical miRNA biogenesis and maturation pathway (Table S1). This analysis included 25,135 genes profiled from 20 disease groups consisting of 139 total excision specimens of cutaneous melanoma in various stages (Clark level I and II-radial growth phase, Clark level III, IV and V-vertical growth phase), melanoma metastases to skin and lymph node, common acquired melanocytic nevi, dysplastic nevi with low and high atypia, normal skin, basal cell carcinoma and squamous cell carcinoma. These two studies used whole genome oligo-microarray platforms: GPL1708 Agilent-012391 Whole Human Genome Oligo Microarray G4112A [21] and GPL570 Affymetrix GeneChip Human Genome U133 Plus 2.0 Array [22]. This analysis examined the 5,023 genes (out of 25,135) significantly altered based on the following criteria: overall gene score (top ranking genes), disease group score (significance among disease groups) and *P*-value specific to the gene and the disease group. For example, Dicer1 ranked among the top 20% most significantly altered genes, appeared in 11 out of 20 disease groups and it was expressed 2.54-fold higher than basal cell carcinoma (*P*-value  = 0.0055) (Table S1). Color specified direction of regulation (red = up, green = down and orange = no direction), while size indicates magnitude of change.

### Cell Lines and Cell Culture

The detailed summary of cell lines is shown (Table S2). TE 354.T cell line was purchased from the American Type Culture Collection (Manassas, VA) and cultured in Dulbecco's modified Eagle's medium with 10% fetal bovine serum (Gibco, Sydney, Australia) and 1% penicillin/streptomycin (Invitrogen). A2058, A375P, C32, A375SM and HEK 293 cells (embryonic kidney 293) were kindly provided by Dr. Stanley N. Cohen, Stanford school of medicine, CA. These cells were cultured in Dulbecco's modified Eagle's medium supplemented with 10% FCS and 2 mM glutamine. WM983A (Coriell), WM278 (Coriell), WM35 and WM1552C were purchased (Wistar institute, Philadelphia, PA) and cultured in 80% MCDB153 (Sigma), 20% Leibovitz's L15 (Mediutech), 2% fetal bovine serum, 5 ug/ml insulin (Sigma) and 1.68 mM CaCl_2_. Three types of epidermal primary melanocytes isolated from 3 individuals with light, medium and dark skin were purchased from ScienCell (Carlsbad, CA) and cultured in melanocyte medium as specified by ScienCell. All cell cultures, except for TE 354.T (at 37°C and 10% CO_2_), were incubated at 37°C in a 5% CO_2_ completely humidified incubator.

### Western Blot Detection of Dicer

Cultured cells were lysed in NP40 Cell Lysis Buffer (Invitrogen, Carlsbad, CA) and spun at 16,000×g to extract soluble proteins. Twenty microgram of total protein was resolved on a 4-20% Tris-Glycine gradient gel (BioRad) and blotted onto a nitrocellulose membrane. The membrane was blocked with 5% powdered nonfat milk in TBST buffer for 1 hour, then incubated with 1∶500 dilution of anti-Dicer or 1∶50,000 dilution of anti-SDHA antibody (Abcam) or 1∶1000 dilution of α-tubulin antibody overnight at 4°C. The membrane was then washed three times with TBST buffer and incubated with anti mouse HRP conjugated secondary antibody, washed thrice with TBST, developed with the ECL Western Blotting Substrate (Pierce, Rockford, IL) and imaged and analyzed on a Kodak Image Station 4000 MM Pro (Carestream Health, Rochester, NY). Relative band intensity for Dicer was normalized against SDHA or α-tubulin as a loading control and quantified according to pixel intensity using Adobe Photoshop.

### Quantitative real-time reverse transcription-PCR (qRT-PCR)

For measuring mRNA levels, total RNA was extracted from cultured cells with TRIzol and the reverse transcription of purified RNA was performed using oligo(dT) priming and superscript II reverse transcription according to the manufacturer's instructions (Invitrogen). The quantification of Dicer and GAPDH transcripts by Real-time quantitative PCR amplification of a cDNA template corresponding to 15 ng total RNA was performed using TaqMan Universal PCR Master Mix and TaqMan gene expressions assay probes (Applied Biosystems, Foster City, CA, USA). As previously described [13], for all miRNAs, 5 ng total RNA was used as a template for TaqMan® miRNA assays (Applied Biosystems, Foster City, CA, USA). All reactions were run in an ABI 7500 Fast system (Applied Biosystems). Cycle threshold (Ct) values for each miRNA were normalized to a small nuclear RNA RNU6 and for Dicer mRNA Ct values were normalized to GAPDH (ΔCt) and represented as 2^−ΔCT^.

## Results

### Dicer Up-regulation in Cutaneous Malignancies Is Specific to Melanoma

**Figure 1:**
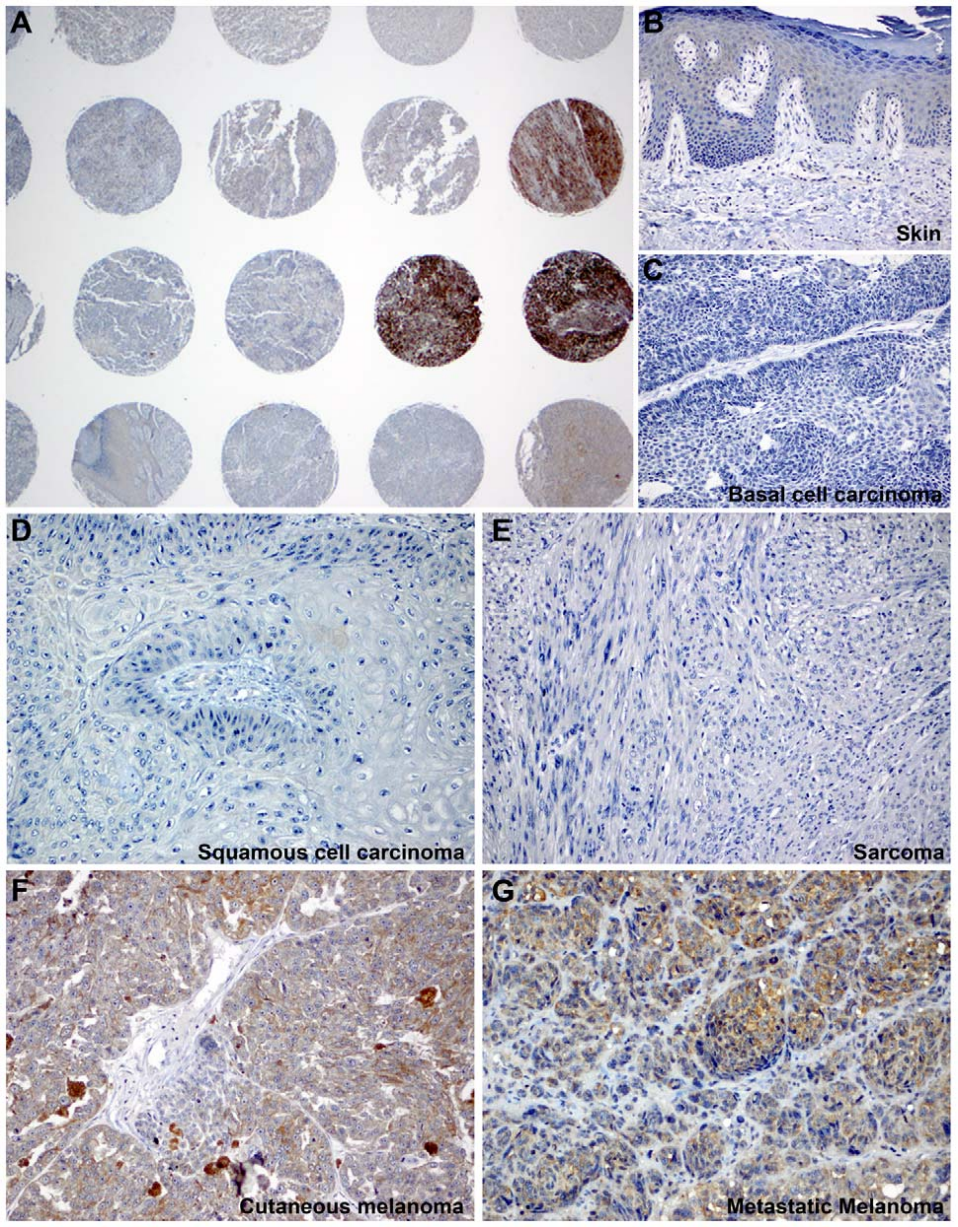
Dicer was specifically expressed in melanoma cells and not carcinoma or sarcoma cells. A) Dicer immunoreactivity was variable across different types of skin cancer by immunohistochemistry using tissue microarrays. B) Normal epidermal keratinocytes exhibited low, basal level of Dicer expression. C–E) The cytoplasm of basal cell carcinoma, squamous cell carcinoma and sarcoma cells were negative for Dicer, respectively. In contrast, melanoma cells strongly and diffusely expressed Dicer in both primary cutaneous (F) and metastatic (G) melanoma. Original magnification: A, 20X; B–C, 100X and D–G, 200X.

**Figure 2:**
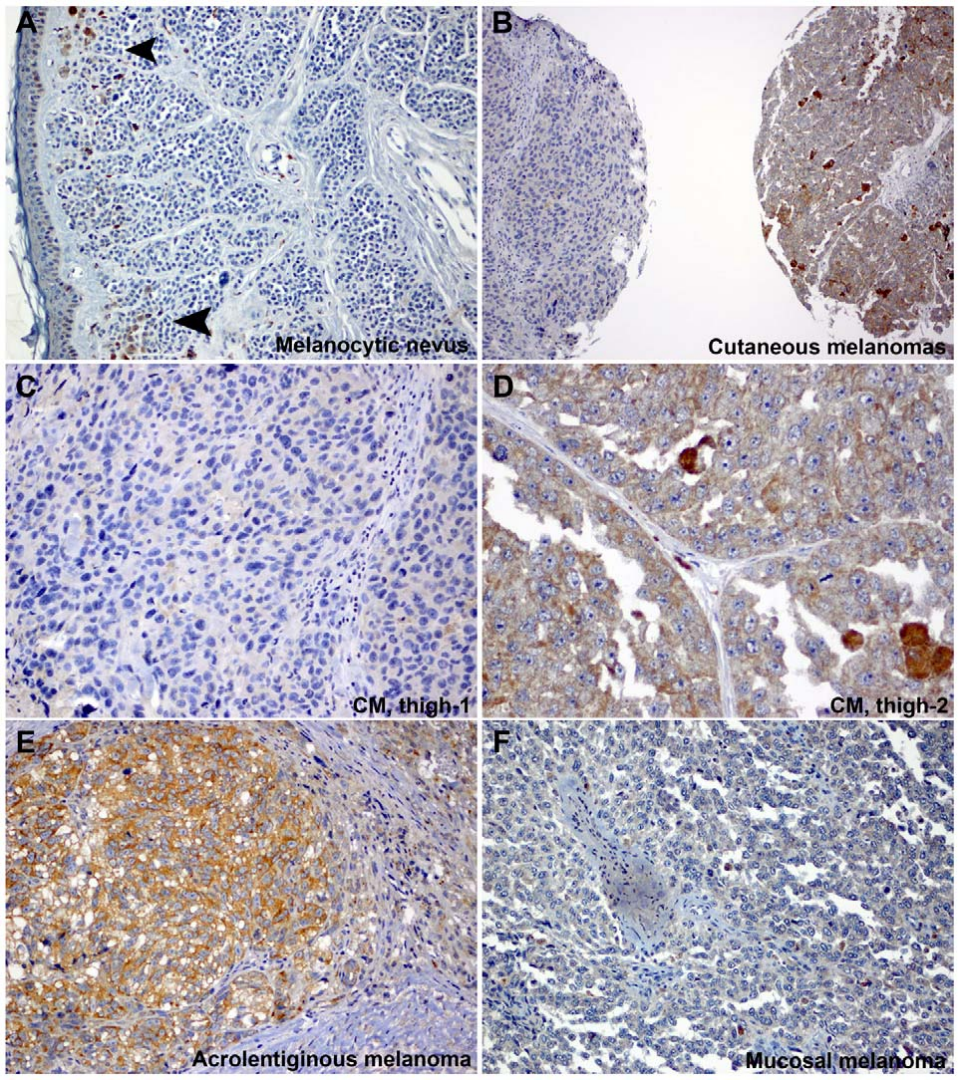
Dicer was expressed at higher levels in cutaneous and acrolentiginous melanomas. A) Intradermal melanocytic nevus cells weakly expressed Dicer in a small group of cells in the superficial dermis (arrowheads) whereas melanoma cells diffusely expressed Dicer at higher levels (B). However, two independent individuals with cutaneous melanoma (CM), both excised from the thigh, expressed Dicer at different levels (B, left and right cores). C–D) Both tissue cores are shown at a higher magnification (CM, thigh-1 and CM, thigh-2). The immunoreactivity for Dicer in melanoma cells was granular and cytoplasmic. E) Cancer cells strongly expressed Dicer in an acrolentiginous melanoma while they were negative for Dicer in a mucosal melanoma (F). Original magnification: A–B, 100X; C, E–F, 200X and D, 400X.

We initially sought to determine if Dicer is expressed in any of the major categories of human cutaneous malignancies, namely melanoma, carcinoma or sarcoma. To this end, we tested a large clinical sample set of formalin-fixed paraffin-embedded (FFPE) benign and malignant tumors (n = 404) including melanocytic nevi (benign melanocytic hyperplasia), a variety of melanoma subtypes (cutaneous, acrolentiginous, mucosal, ocular and desmoplastic), a variety of carcinomas (squamous, basal cell and eccrine) and sarcomas (Table 1) using a monoclonal anti-human Dicer antibody and immunohistochemistry on tissue microarrays (TMAs) (Fig. 1A) and full tumor sections (Fig. S1A-F). After determining the immunostaining pattern for Dicer in normal skin and other organs, we established a semi-quantitative, four-point ordinal immunoreactivity scoring scale: negative (0), weakly positive (1), moderately positive (2) and strongly positive (3). Scores for multiple cores from one case were averaged, and final Dicer scores were categorized into a three-level grouping of Negative, Low (>0 and ≤1.5) or High (>1.6) for analyses that included all 404 patients (Table 1). In normal skin, epidermal keratinocytes expressed Dicer consistently at low levels (Fig. 1B). Carcinoma cells of skin (basal and squamous), adenocarcinoma cells (primary or metastatic) and sarcoma cells expressed none to very low levels of Dicer (Fig. 1C-E). In contrast, the cytoplasm of primary cutaneous and metastatic melanoma cells exhibited high levels of Dicer immunoreactivity (Fig. 1F-G). The majority of carcinomas (94.5%), sarcomas (100%) and tumors with neural differentiation (100%) of the skin was negative for Dicer or expressed it at low levels (Table 1). Among tumors with melanocytic differentiation, we observed low levels of Dicer immunoreactivity in the cytoplasm of melanocytic nevus cell (Fig. 2A) compared to the high levels seen in cutaneous and acrolentiginous melanomas (Fig. 2B-E). The cytoplasm of mucosal melanoma cells was devoid of Dicer immunoreactivity (Fig. 2F). Dicer was consistently expressed at high levels in the majority of metastatic (55.8%), acrolentiginous (55.0%) and cutaneous (46.4%) melanomas compared to mucosal melanoma (12.5%), conferring further cancer-cell specificity among different types of melanomas (Table 1). The remainder of Dicer-positive cutaneous melanomas expressed Dicer either at low levels (34.7%) or exhibited no expression (18.9%). Two different patients with cutaneous melanoma (both excised from the thigh) exemplified this variability in expression Dicer. The melanoma from patient 1 expressed Dicer at low levels (Fig. 2B and C) compared to that in patient 2 who expressed Dicer at high levels (Fig. 2B and D). Dicer expression was not associated with differences in gender (n = 328) or age (n = 335) in the examined cutaneous tumors (Table S3). Among the cutaneous (n = 93) and acrolentiginous (n = 40) melanomas examined, there was no statistically significant association between Dicer expression and the anatomic site (head and neck, trunk, upper and lower extremities or genital skin) (Table S4).

**Table 1:** Dicer expression in relation to tumor type (n = 404).

		Dicer Immunoreactivity									
			Negative		Low (≤1.5)		High (>1.6)		*P*-Value^1^	Mean ± SD	*P*-Value
		Total	n	%	n	%	n	%			
**Tumor Type**	Normal Tissue	12	11	91.7%	1	8.3%	0	-		0.04±0.14	
	Melanocytic Nevus	71	13	18.3%	48	67.6%	10	14.1%		0.83±0.60	
	Cutaneous Melanoma	95	18	18.9%	33	34.7%	44	46.4%		1.40±0.96	
	Acrolentiginous Melanoma	40	4	10.0%	14	35.0%	22	55.0%		1.61±0.92	
	Mucosal Melanoma	24	8	33.3%	13	54.2%	3	12.5%		0.79±0.65	
	Ocular Melanoma	4	0	-	2	50.0%	2	50.0%		1.50±0.58	
	Desmoplastic Melanoma	8	5	62.5%	2	25.0%	1	12.5%		0.44±0.73	
	Metastatic Melanoma	52	2	3.8%	21	40.4%	29	55.8%		1.71±0.83	
	Carcinoma	73	45	61.6%	24	32.9%	4	5.5%		0.39±0.59	
	Sarcoma	12	11	91.7%	1	8.3%	0	-		0.08±0.29	
	Neural Tumors	13	9	69.2%	4	30.8%	0	-	**<0.0001**	0.23±0.39	<**0.0001** 2

1 Pearson Chi-Square test for proportions.

2 Kruskal-Wallis (k = 3 or more) non-parametric test for continuous values.

**Figure 3:**
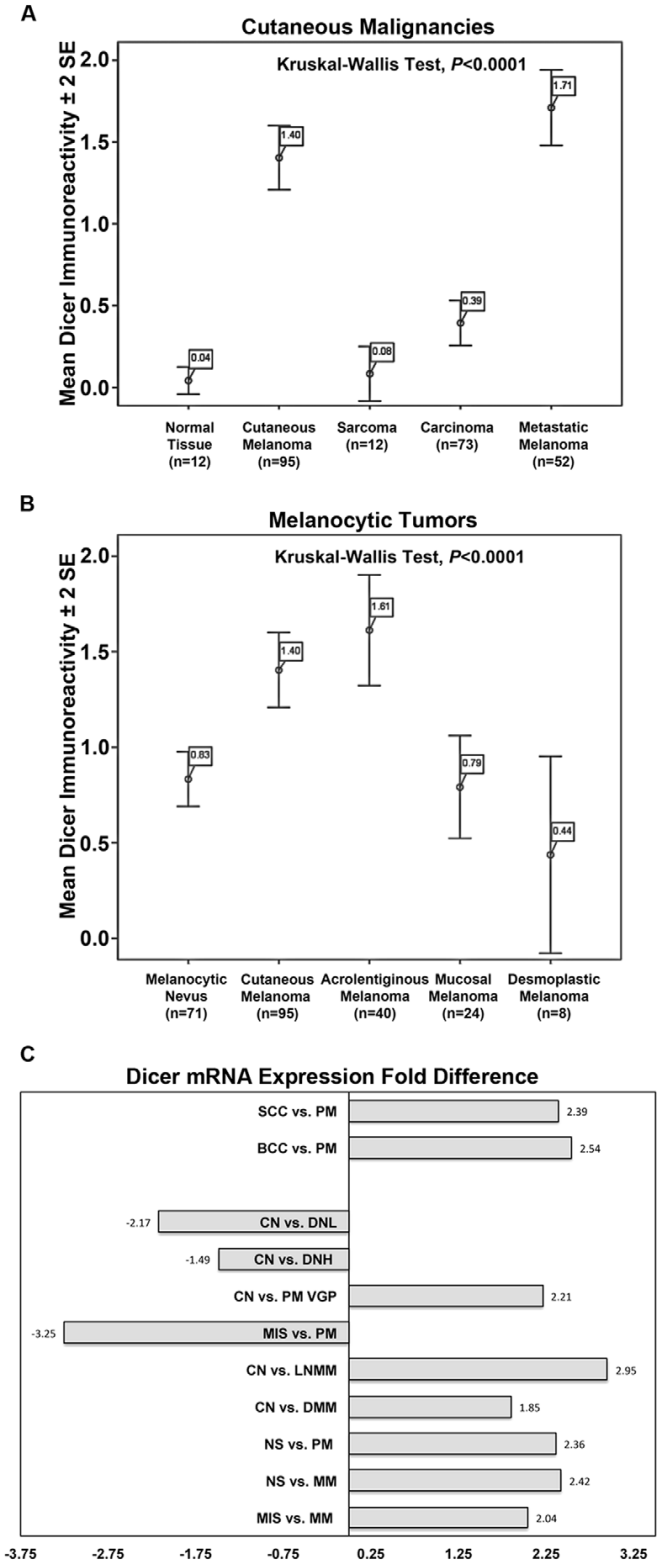
Dicer expression was cancer-cell specific among cutaneous malignancies and was significantly higher in primary and metastatic melanoma compared to common melanocytic nevus. A) Primary cutaneous (n = 95) and metastatic (n = 52) melanomas had the highest levels of Dicer immunoreactivity vs. carcinomas (n = 73) and sarcomas (n = 12). B) Cutaneous (n = 95) and acrolentiginous (n = 40) melanomas had the highest levels of Dicer immunoreactivity vs. melanocytic nevi (n = 71), mucosal (n = 24) and desmoplastic (n = 8) melanomas. Dicer immunoreactivity is shown as mean (boxed) ±2 standard error (SE). The statistical significance was measured for all independent samples comparing to each other (Kruskal-Wallis Test, *P*<0.0001). C) Pooled analysis performed on publically available transcriptional profiling data showed significant changes in Dicer mRNA levels during melanoma progression. This analysis included 25,135 genes from 20 disease groups and 139 individual specimens of squamous cell carcinoma (SCC), basal cell carcinoma (BCC), primary melanoma (PM), common nevus (CN), dysplastic nevus with low (DNL), dysplastic nevus with high atypia (DNH), primary melanoma vertical growth phase (PM VGP), melanoma in situ (MIS), lymph node melanoma metastases (LNMM), dermal melanoma metastases (DMM), normal skin (NS) and melanoma metastases (MM) [21], [22]. Dicer1 ranked among the top 20% most significantly altered genes.

Overall, when compared among all examined cutaneous malignancies, Dicer up-regulation was tumor-type specific by immunostaining, as Dicer was highly expressed by melanomas (metastatic and cutaneous) compared to carcinomas or sarcomas (*P*<0.0001, Fig. 3A). Furthermore Dicer up-regulation was specific to the melanoma subtype, i.e. cutaneous and acrolentiginous compared to mucosal and desmoplastic melanomas (*P*<0.0001, Fig. 3B). Importantly, higher Dicer levels were detected in cutaneous, acrolentiginous and metastatic melanomas compared to common melanocytic nevi. To confirm the up-regulation of Dicer in melanoma, we performed a pooled analysis by mining published whole genome oligo-microarray dataset on two recent large studies that profiled gene expression pattern in excisional specimens of cutaneous tumors (n = 139) [21], [22]. The combined dataset included 20 different disease groups consisting of cutaneous melanoma in various stages (in situ, Clark level I and II-radial growth phase, Clark level III, IV and V-vertical growth phase), melanoma metastases to skin and lymph node, common acquired melanocytic nevi, dysplastic nevi with low and high atypia, normal skin, basal cell carcinoma and squamous cell carcinoma (Table 2). The combined dataset provided 25,135 genes, which we interrogated for Dicer mRNA expression levels. Comparing cutaneous melanoma to other skin cancers, squamous and basal cell carcinoma, or normal skin (mostly consisting of keratinocytes) showed significantly higher levels Dicer mRNA in melanoma (Fig. 3C, Table 2), confirming our immunostaining results (Fig. 3A) and indicating an up-regulation at the level of mRNA accumulation. Furthermore, invasive and metastatic melanomas had significantly higher levels of Dicer mRNA than common melanocytic nevi (Fig. 3C, Table 2), again confirming our immunostaining results (Fig. 3B). Interestingly, the Dicer mRNA levels are decreased in dysplastic when compared common melanocytic nevi and in melanoma in situ when compared to invasive (Fig. 3C, Table 2). Overall, these results showed that Dicer up-regulation, at both the protein and the RNA levels, is specific to melanoma subtypes and that Dicer levels are higher in primary cutaneous and metastatic melanomas compared to common melanocytic nevi.

**Table 2:** Pooled analysis performed on Dicer mRNA expression in publically available gene expression profiling studies (clinical sample size = 139, disease groups = 20).

	DICER1	
	*P*-value	Fold change
**NS vs. BCC**	NS	NS
**NS vs. SCC**	NS	NS
**SCC vs. BCC**	NS	NS
**SCC vs. PM**	0.0108	+2.39
**BCC vs. PM**	0.0055	+2.54
**CN vs. DNL**	0.0006	−2.17
**CN vs. DNH**	0.0074	−1.49
**CN vs. C I RGP**	NS	NS
**CN vs. C II RGP**	NS	NS
**CN vs. C III VGP**	NS	NS
**CN vs. C IV VGP**	NS	NS
**CN vs. C V VGP**	0.0323	+2.21
**MIS vs. PM**	0.0391	−3.25
**CN vs. LNMM**	0.0244	+2.95
**CN vs. DMM**	0.0242	+1.85
**NS vs. MIS**	NS	NS
**NS vs. PM**	0.0082	+2.36
**NS vs. MM**	0.0045	+2.42
**MIS vs. MM**	1.70E-06	+2.04
**PM vs. MM**	NS	NS

To stay consistent with the direction of disease progression, the sign (+ or −) was changed appropriately. Fold changes with a (−) sign are lower and those with a (+) sign are higher than the disease group to which it was compared. For example, PM showed 2.36-fold increase in Dicer mRNA levels when compared to NS. The abbreviations of disease groups are as follows: DNL-dysplastic nevus low-grade atypia; DNH-dysplastic nevus high-grade atypia; CN-common nevus; C I RGP-Clark's level I radial growth phase; C II RGP-Clark's level II radial growth phase; C III VGP-Clark's level III vertical growth phase; C IV VGP-Clark's level IV vertical growth phase; C V VGP-Clark's level V vertical growth phase; LNMM-lymph node metastatic melanoma; DMM-dermal metastatic melanoma; MIS-melanoma in situ; PM-primary melanoma invasive; MM-metastatic melanoma; NS-normal skin; BCC-basal cell carcinoma; SCC-squamous cell carcinoma. NS-not significant.

### Clinical Features Associated with Dicer Up-regulation

**Figure 4:**
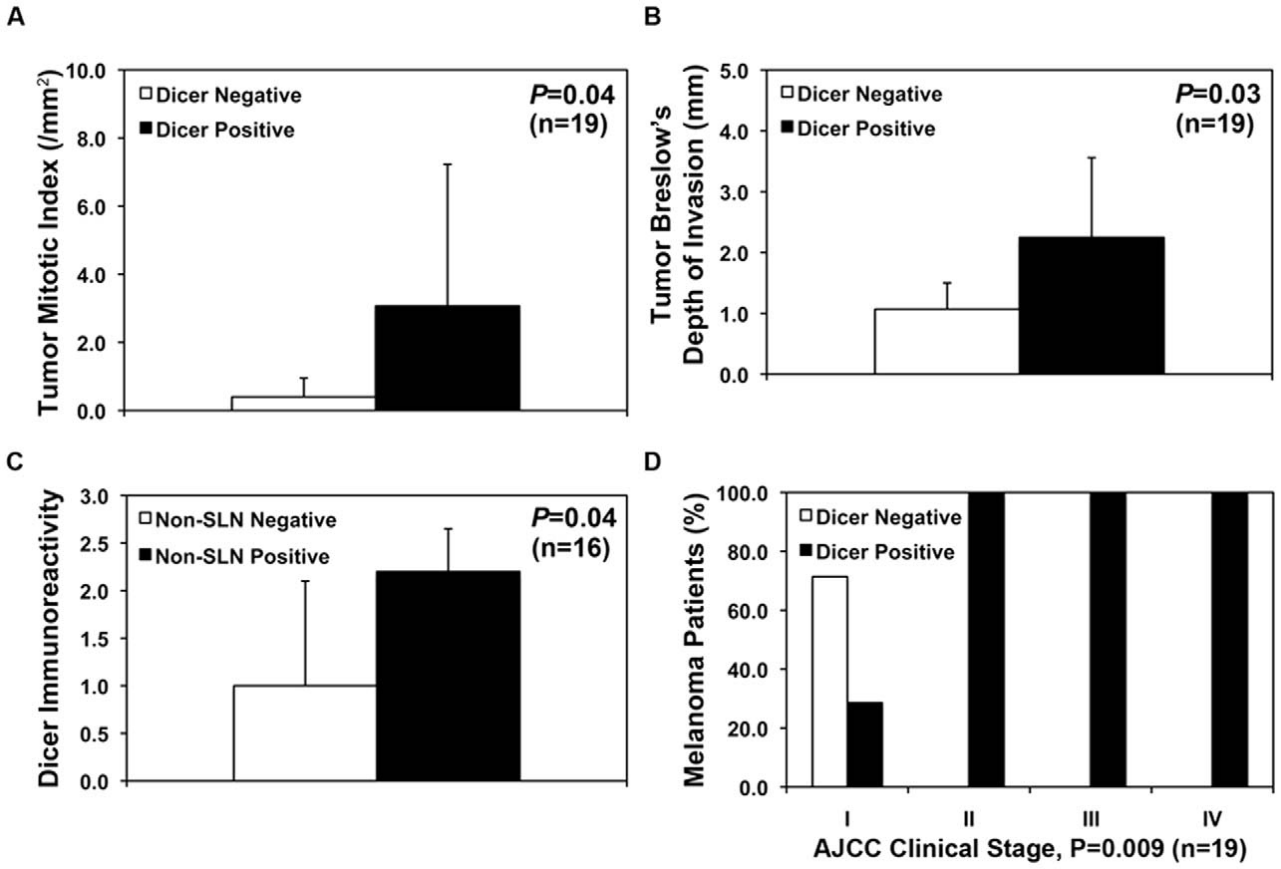
Dicer expression significantly correlated with clinical outcome in patients with cutaneous melanoma. A–B) The mean values for melanoma mitotic index (per mm^2^) and Breslow's depth of invasion significantly correlated with Dicer expression (*P* = 0.04 and *P* = 0.03, respectively). C) Dicer immunoreactivity score was significantly associated with metastasis to non-sentinel lymph node (SLN) in patients with cutaneous melanoma (*P* = 0.04). D) The American Joint Commission on Cancer (AJCC) staging was significantly associated with Dicer expression (*P* = 0.009).

To characterize Dicer expression pattern and distribution, we immunostained melanocytic nevi, cutaneous and metastatic melanomas in complete sections (n = 33). In 30% of cutaneous melanomas, the intratumoral expression of Dicer varied where immunoreactivity was focally high compared no expression in other areas within the same lesion (Fig. S1A). Dicer was expressed both in the intraepidermal (*in-situ*) and dermal (invasive) melanoma cells (Fig. S1B-D). The majority of cutaneous melanomas, TMAs and complete sections, expressed Dicer either at high (44 of 95, 46.3%) or low (33 of 95, 34.7%) levels; however a minority of the cases (18 of 95, 18.9%) was negative for expression. This finding prompted us to investigate whether this difference might be associated with clinical outcome in cutaneous melanomas patients with available clinical information. For 19 patients, tumor pathological features, clinical stage and clinical follow-up (rage 7.1 to 69.7 months; median = 22.4 months; mean = 26.6 months) were available. Dicer expression level was significantly associated with tumor mitotic index (*P* = 0.04, n = 19) and Breslow's depth of invasion (*P* = 0.03, n = 19) (Fig. 4A-B-D, Tables 3-4), two of three most important parameters currently used in staging and predicting prognosis for melanoma patients by the American Joint Committee on Cancer (AJCC) [19]. Dicer expression was not significantly associated with other tumor pathological features such as histological subtype, inflammation (tumor infiltrating lymphocytes), regression, ulceration or Clark's level (Table 4). Most important, Dicer expression significantly correlated with metastasis to the non-sentinel lymph node (SLN, *P* = 0.04, n = 16) (Fig. 4C, Table 4) and the AJCC clinical stage (*P* = 0.009, n = 19) (Fig. 4D). Melanoma patients with positive Dicer expression did demonstrate a trend towards higher rates of SLN, organ and distant metastases (Table 4); however this association did not reach a statistical significance.

**Table 3:** Dicer expression in relation to melanoma mitotic activity and depth of invasion (n = 19).

	Dicer Immunoreactivity		
	Negative n = 5	Positive n = 14	*P*-Value^1^
	Mean ± SD	Mean ± SD	
**Mitotic Index**	0.40±0.55	3.07±4.16	**0.04**
**Breslow's Depth (mm)**	1.07±0.43	2.25±1.31	**0.03**
**Clark's Level**	3.20±0.84	3.79±0.43	0.08

1 Mann-Whitney (k = 2) non-parametric test for continuous values.

**Table 4:** Dicer expression in relation to melanoma clinicopathological features (n = 19).

		Dicer Immunoreactivity						
		Negative		Positive		*P*-Value^1^	Mean ± SD	*P*-Value
		n	%	n	%			
**Subtype**	Superficial spreading	5	29.4%	12	70.6%		1.35±0.99	
	Nodular	0	-	2	100%	0.99	2.00±1.41	0.43^2^
**Tumor infiltrating lymphocytes**	Absent	2	28.6%	5	71.4%		1.43±1.13	
	Present	3	25.0%	9	75.0%	0.99	1.41±0.99	0.96^2^
**Regression**	Absent	4	25.0%	12	66.7%		1.50±1.03	
	Present	1	50.0%	1	50.0%	0.49	0.50±0.71	0.18^2^
**Ulceration**	Absent	5	38.5%	8	61.5%		1.23±1.09	
	Present	0	-	6	100%	0.13	1.83±0.75	0.28^2^
**Stage**	I	5	71.4%	2	28.6%		0.71±1.25	
	II	0	-	4	100%		1.75±0.50	
	III	0	-	2	100%		1.50±0.71	
	IV	0	-	6	100%	**0.009**	2.00±0.63	0.17^3^
**Sentinel Lymph Node Metastases**	Negative	5	38.5	8	61.5%		1.23±1.09	
	Positive	0	-	6	100%	0.12	1.83±0.75	0.28^2^
**Non-Sentinel Node Metastases** 4	Negative	5	45.5%	6	54.5%		1.00±1.10	
	Positive	0	-	5	100%	0.12	2.20±0.45	**0.04** 2
**Organ Metastases**	Absent	5	33.3%	10	66.7%		1.33±1.11	
	Present	0	-	4	100%	0.53	1.75±0.50	0.52^2^
**Distant Metastases**	M0	5	38.5%	8	61.5%		1.15±1.07	
	M1	0	-	6	100%	0.13	2.00±1.41	0.10^2^
**Vital Status**	Alive	0	-	4	100%		1.75±0.50	
	Dead	5	33.3%	10	66.7%	0.53	1.33±1.11	0.52^2^

1 Pearson Chi-Square test for Stage and Tumor Thickness (Table 3); Fisher's Exact Test for all other variables.

2 Mann-Whitney (k = 2) non-parametric test for continuous values.

3 Kruskal-Wallis (k = 3 or more) non-parametric test for continuous values.

4 Three cases with unknown nodal status.

### Expression of Dicer and let-7 miRNA family *in vitro*

**Figure 5:**
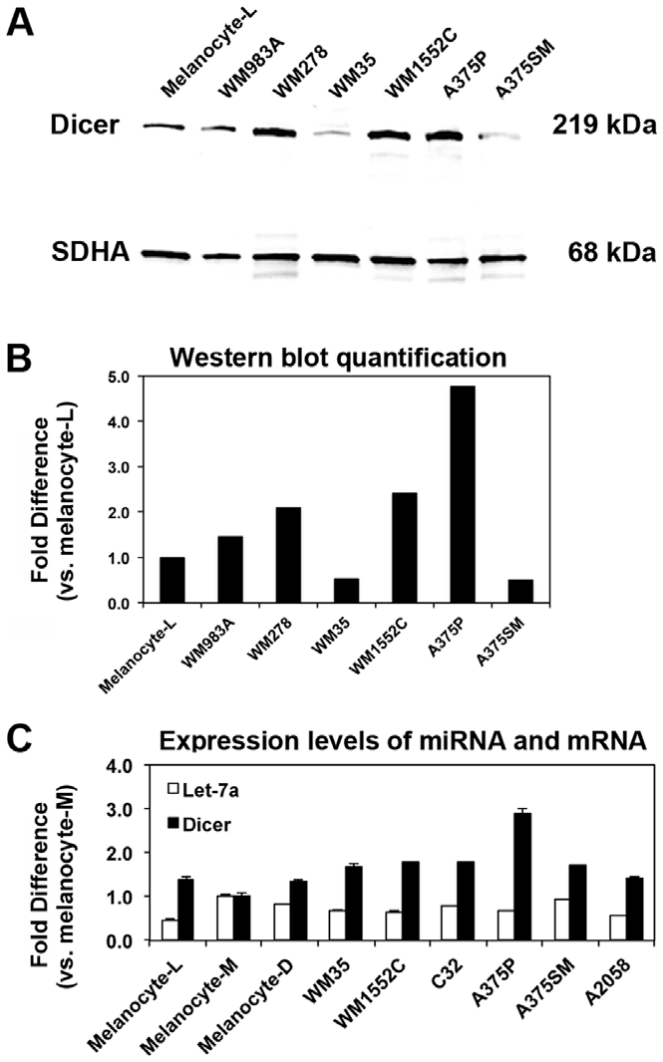
Dicer expression in cell lines recapitulated the observed deregulation in clinical specimens. A) Western blot analysis of Dicer shows a 219-kDa band. Relative band intensity was compared to succinate dehydrogenase (SDHA, 68 kDa) as a loading control. B) Western blot quantification showed >2 to 4-fold change in Dicer levels in melanoma cell lines (WM278, WM1552C and A375P) when compared to melanocyte-L or other melanoma cell lines (WM35 and A375M). C) Dicer mRNA expression did not correlate with mature let-7a expression *in vitro*. Using qRT-PCR, the relative expression levels of let-7a miRNA and Dicer mRNA were compared to show no significant correlation. All qRT-PCRs were performed in triplicates. Data were normalized to small nuclear RNA RNU6 for let-7a and GAPDH mRNA for Dicer. The samples are: Primary melanocytes were cultured from individuals with light (Melanocyte-L), medium (Melanocyte-M) and dark (Melanocyte-D) skin color, WM983A (primary melanoma), WM278 (primary melanoma), WM35 (primary melanoma), WM1552C (primary melanoma), C32 (amelanotic primary melanoma), A375P (metastatic melanoma), A375SM (metastatic melanoma) and A2058 (metastatic melanoma).

Our findings in clinical melanoma specimens raised the question of whether Dicer up-regulation might be intrinsic to the tumor cells. We compared Dicer protein levels between primary melanocytes, primary (n = 3) and metastatic (n = 3) melanoma cell lines. Western blot analysis combined with measured relative band intensity, normalized against succinate dehydrogenase (SDHA), showed >2 to 4-fold higher Dicer levels in melanoma cell lines (WM278, WM1552C and A375P) when compared to melanocyte-L or other melanoma cell lines (WM35 and A375M) (Fig. 5A-B). Furthermore, we tested additional cell lines; combined western blot analysis and measured relative band intensity showed >2-fold higher Dicer levels in melanoma cell lines (WM1552C and A2058) compared to basal cell carcinoma (BCC), primary melanocytes (n = 3) or other melanoma cell lines (WM35 and C32) (results not shown). The melanocytes were derived from three different individuals with light, medium and dark skin color. Dicer levels were comparable among the three melanocytes, despite the skin color. Overall, Dicer expression in cell lines recapitulated the observed deregulation in clinical specimens, confirming higher Dicer immunostaining in melanomas when compared to melanocytic nevi or carcinomas of the skin (Figs. 1, 2 and 3).

Given the differential levels of Dicer expression in melanoma cell lines and the prior finding of let-7a miRNA exerting a negative feedback loop on Dicer expression in lung and pancreatic cell lines [23], it was conceivable that the differences in let-7a miRNA levels might be associated with Dicer regulation via such mechanism in melanoma. We compared the relative expression of the entire let-7 family to Dicer mRNA levels using quantitative real-time reverse transcription-PCR (qRT-PCR). Neither Dicer mRNA nor Dicer protein levels correlated with any of the mature miRNAs tested (let-7a, let-7b, let-7c, let-7d, let-7e, let-7f, left-7g or let-7i). Interestingly, we found that let-7b expression levels are significantly down-regulated in metastatic (A375P, A375SM and A2058) compared to primary (WM35, WM1552C and C32) melanoma cell lines; while let-7d levels are significantly down-regulated in all six melanoma cell lines (metastatic and primary) compared to primary melanocytes (Fig. S2).

### Perturbed miRNA Biogenesis Pathway during Melanoma Progression

**Figure 6:**
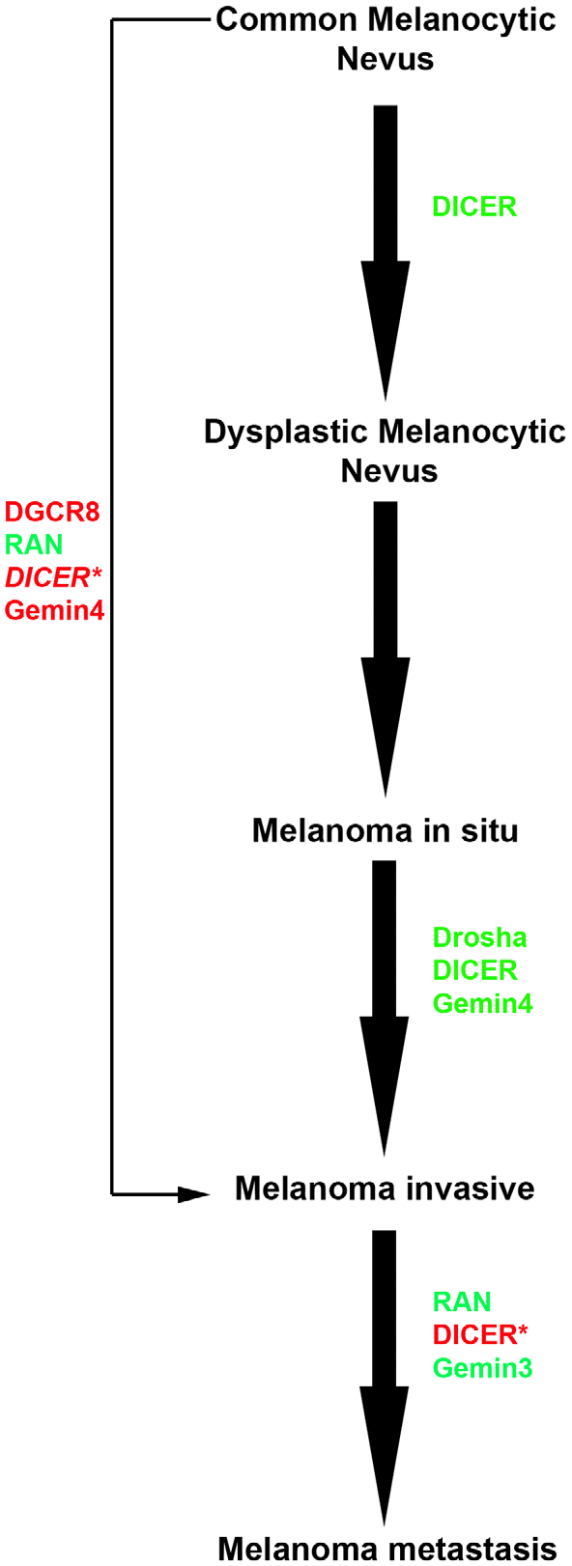
Enzymes involved in the canonical miRNA biogenesis are deregulated during melanoma progression. Combined Dicer immunoreactivity, presented herein (denoted by asterisk ‘*’), and mRNA transcriptional profiling [21], [22] examined for Dicer and other enzymes in the miRNA biogenesis showed a global change in their expression levels during tumor progression. Enzymes shown in red are up-regulated and those in green are down-regulated. Up-regulation of Dicer (italicized) from common melanocytic nevus to invasive melanoma was found both in our study and others [21], [22]. Dicer, DGCR8 and Gemin4 ranked among the top 20 percentile of most significantly altered genes.

Since the expression of Dicer is significantly altered from common to dysplastic nevi to melanoma in situ to invasive and to metastatic melanoma, we interrogated the same combined dataset, which included 20 different disease groups and 25, 135 genes, for the mRNA levels of all the known enzymes involved in canonical miRNA biogenesis by performing a pooled analysis mining published whole genome oligo-microarray dataset [21], [22]. Enzymes tested in the canonical miRNA biogenesis pathway included Drosha, DGCR8, RAN, XPO5, Dicer1, GEMIN3, GEMIN4, EIF2C2, Ago2 and TRBP (Table S1). Dicer1, DGCR8 and Gemin4 ranked among the top ∼20 percentile of most significantly altered genes. We represent this data with respect to a linear, step-wise progression model for melanomagenesis, plotting changes in the expression level of the enzyme detected in our own immunostaining (protein) and in microarray pooled analysis (mRNA) (Fig. 6). This analysis suggests that the biogenesis of mature miRNAome maybe enhanced in the early steps of melanocyte transformation and melanoma formation raising the possibility that Dicer may play a central role in the melanocyte transformation and metastasis. Surprisingly, Dicer, Drosha and Gemin4 are down-regulated in melanoma in situ compared to invasive melanoma; in addition, Dicer is down-regulated in dysplastic nevi compared to common nevi, suggesting a global repression of miRNA biogenesis in these steps.

## Discussion

In this study, we examined the expression and the clinical relevance of Dicer in cutaneous melanoma. We showed that a large portion of cutaneous melanomas exhibited up-regulation of Dicer significantly associated with aggressive cancer features. We demonstrated definitive evidence that Dicer up-regulation is specific to the malignant proliferation of melanocytes (melanoma) and not keratinocytes (carcinoma) or fibroblasts (sarcoma) in 404 human skin tumors. Given that the “melanoma disease group” is a heterogeneous cancer, to have a complete representation, we compared Dicer expression among the various subtypes of melanomas occurring in glabrous (subungual, palm and sole) skin, non-glabrous skin, eye, mucosal sites (e.g. oral, urothelial and anal mucosa) and metastatic sites (variety of organs) to melanocytic nevi. These immunostaining results clearly showed that Dicer up-regulation was specific to cutaneous, acrolentiginous and metastatic melanomas. To complement the immunostaining, we carried out a pooled analysis using two recent large studies that profiled gene expression pattern in cutaneous tumors. This analysis corroborated our immunostaining data and indicated that at least a component of Dicer up-regulation in melanoma is due to differences in mRNA accumulation.

Although our results provide strong evidence that up-regulated Dicer was tumor-cell specific and reflective of mRNA levels, these results provided little mechanistic explanation. Given the involvement of Dicer, Dicer products, and associated components of the RNAi machinery in diverse cellular processes [24] there are certainly numerous mechanisms both for the potential regulation of Dicer in melanoma and for the effects of this regulation in the context of the tumor. Further analysis of both questions will certainly be warranted. Here, we provide a number of (not necessarily exclusive) connections between Dicer functions and other factors that may regulate or depend on these.

First, the presence of cell-specific transcription factors might be expected to partially explain the difference in Dicer expression between melanocytes vs. keratinocytes or fibroblasts. Recently, it has been shown that Dicer is a direct transcriptional target of microphthalmia-associated transcription factor (MITF); tissue-restricted master transcriptional regulator of melanocytes; and that targeted KO of Dicer is lethal to melanocytes [25]. MITF targeting Dicer in melanocytes may partially explain our findings of up-regulated Dicer in melanoma and not carcinoma or sarcoma. As for the underlying mechanisms of increased Dicer expression in melanoma, one class of conceivable mechanisms would occur if human *Dicer* were amplified through gains in DNA copy number by genomic instability. *Dicer* is mapped to chromosome 14 (14q32.13); a genetic locus altered in other human malignancies: esophageal carcinoma [26], nasopharygeal carcinoma [27] and urothelial carcinoma [28]. From a set of primary melanoma cell lines, *Dicer1* locus showed 19.6% gain and 8.7% loss of DNA copy number [29]. Finally, it has been demonstrated that let-7a enforces a negative feedback loop on Dicer expression in lung and pancreatic carcinoma cell lines [23]. Despite the potential of this feedback loop, our measurements of levels for let-7a (or other members of let-7 family) and Dicer do not support a let-7 regulatory loop as the key element of Dicer up-regulated expression in melanoma cell lines.

We found that the expression of Dicer was variable among cutaneous melanomas (n = 95) where, the great majority (81%) of cases expressed it while 19% of cases demonstrated an absence of immunoreactivity. Postulating that this difference could be clinically relevant, we examined correlations with other clinical features, observing a statistically significant association between Dicer expression and melanoma mitotic index and Breslow's depth of invasion, both indicative of a more aggressive cancer (these are two of the three most important AJCC staging parameters) currently used to determine prognosis for melanoma patients [19]. Dicer expression significantly correlated with non-SLN metastasis and AJCC stage but not disease-specific survival. Given the small patient population with available clinical follow-up information in this study (n = 19), our findings need to be validated in larger melanoma cohorts. Our results suggest analogy to prostate adenocarcinoma where up-regulated Dicer correlated with metastasis to regional lymph nodes and clinical stage [16]. Deregulation of Dicer, or other enzymes in the miRNA biogenesis pathway, maybe a common central feature shared by several solid cancers [16], [17], [18], [30], [31], [32], [33], [34] to globally regulate the biogenesis of oncomirs. From our pooled analysis focusing on all known enzymes that participate in the biogenesis and maturation of canonical miRNAs, we also propose the possibility of a more general phenomenon where several deregulated RNAi enzymes, in addition to Dicer, may influence the various steps in melanoma progression (Fig. 6).

Overall, our results show definitive up-regulation of Dicer in cutaneous melanoma, compared to other skin cancer types, which correlated with a more aggressive behavior. When confirmed by independent studies in larger cohorts, increased Dicer expression may serve as a clinically useful prognostic biomarker for cutaneous melanoma patients. Beyond this, a combined understanding of deregulated Dicer and its influence on the expression pattern of mature miRNAs may lead to indications of directions in which small RNA modulations may contribute therapeutically in melanoma treatment. During the revision of this manuscript, we noted an abstract for a small pilot study [35] comparing Dicer immunostaining pattern among cutaneous melanomas, melanocytic nevi and dysplastic nevi. The abstract suggested that a significantly higher Dicer immunostaining was detected in melanoma cells than in nevus cells.

## Supporting Information

Figure S1 Expression of Dicer in primary cutaneous and metastatic melanomas by immunohistochemistry using complete tumor sections. A) Cancer cells focally expressed Dicer at high levels in the left margin (arrowhead) compared to the cancer cells in the center (asterisks) that were negative for Dicer in the same cutaneous melanoma (CM). B) In another CM, cancer cells expressed Dicer along the dermal-epidermal junction and follicular epithelium (*in situ*, arrowhead) as well as in the dermis (invasive, arrow). C) In an ulcerated CM, cancer cells, invading throughout the dermis, strongly and diffusely expressed Dicer. D) Cancer cells expressed Dicer in *in situ* and invasive components of another CM. E) Melanoma cells expressed Dicer in a subcapsular (arrowhead) location in the sentinel lymph node (SLN) of a patient with metastatic melanoma (MM) compared to the adjacent nodal tissue containing mature lymphocytes (asterisk) that are negative for Dicer. F) In another patient with MM, cancer cells strongly and diffusely expressed Dicer in the SLN, where expanding tumor nodules obliterated the normal lymph node architecture. Under higher magnification, Dicer was localized to the cytoplasm of melanoma cells with a granular quality (inset D and F). Original magnification: A, 200X; B-D, 100X, E, 200X and F, 100X; insets: 400X.
(TIF)

Figure S2 Dicer mRNA expression did not correlate with the expression of any mature miRNA members in the let-7 family *in vitro*. Using qRT-PCR, the relative expression levels of let-7b, let-7c, let-7d, let-7d, let-7f and let-7g miRNAs and Dicer mRNA were compared to show no significant correlation. However, let-7b expression is significantly down-regulated in all three metastatic compared to three primary melanoma cell lines; whereas let-7d expression is significantly down-regulated in all 6 metastatic and primary melanoma cell lines compared to three melanocytes. All qRT-PCRs were performed in triplicates. Data were normalized to small nuclear RNA RNU6 for let-7 family and GAPDH mRNA for Dicer.
(TIF)

Table S1 Pooled analysis performed on enzymes in miRNA biogenesis pathway in publically available gene expression profiling studies (clinical sample size = 139, disease groups = 20).
(DOCX)

Table S2 Summary of cell lines, source and type.
(DOCX)

Table S3 Dicer expression in relation to cutaneous tumors, sex (n = 328) and age (n = 335).
(DOCX)

Table S4 Dicer expression in relation to melanoma type and anatomic site (n = 133).
(DOCX)
