# Supplementary figures and images for: Up-Regulated Dicer Expression in Patients with Cutaneous Melanoma
Source: PLoS One. 2011 Jun 17;6(6):e20494. doi: 10.1371/journal.pone.0020494 (PMC3117784; doi:10.1371/journal.pone.0020494)

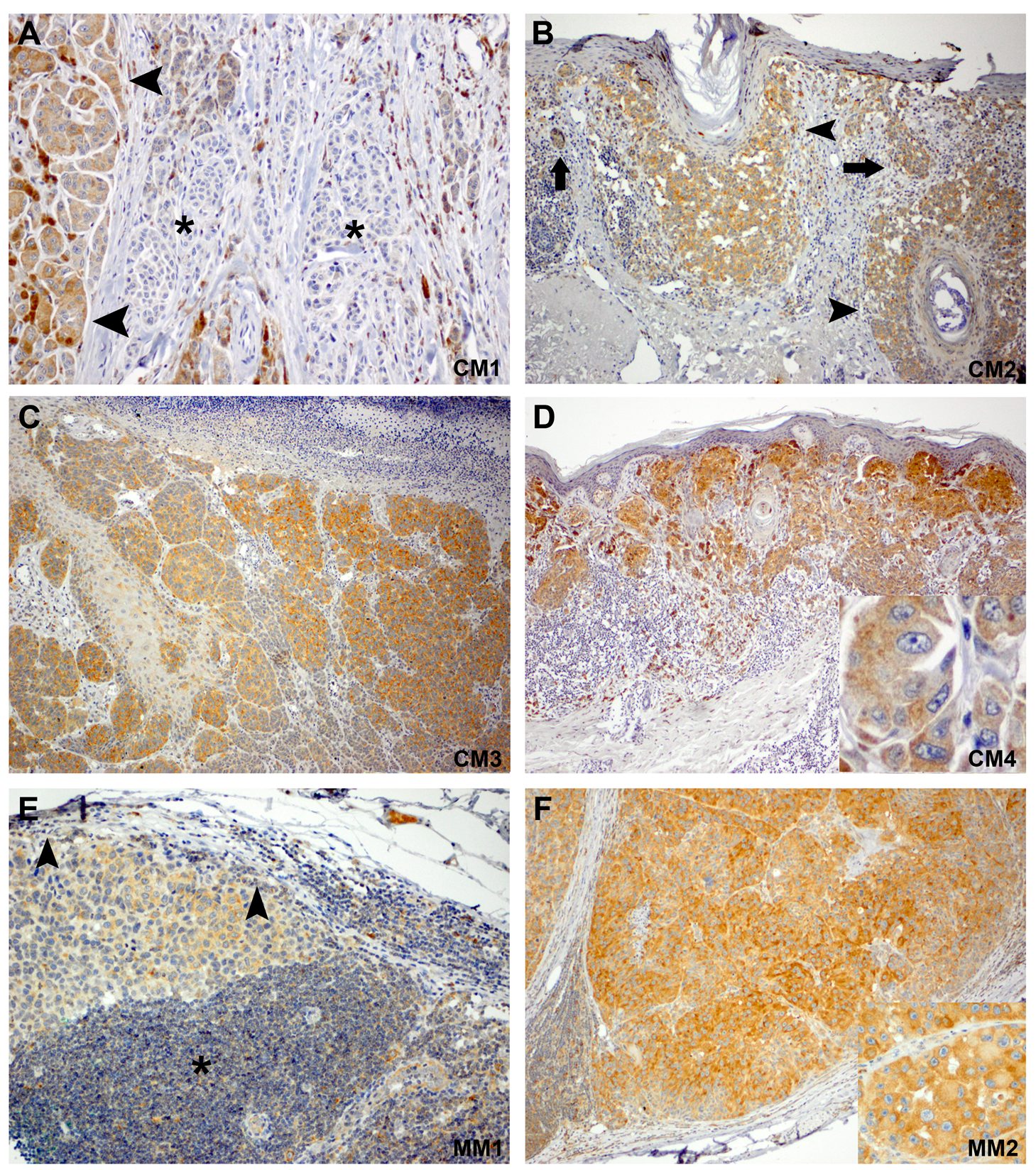

Supplement: Figure S1 — Expression of Dicer in primary cutaneous and metastatic melanomas by immunohistochemistry using complete tumor sections. A) Cancer cells focally expressed Dicer at high levels in the left margin (arrowhead) compared to the cancer cells in the center (asterisks) that were negative for Dicer in the same cutaneous melanoma (CM). B) In another CM, cancer cells expressed Dicer along the dermal-epidermal junction and follicular epithelium (in situ, arrowhead) as well as in the dermis (invasive, arrow). C) In an ulcerated CM, cancer cells, invading throughout the dermis, strongly and diffusely expressed Dicer. D) Cancer cells expressed Dicer in in situ and invasive components of another CM. E) Melanoma cells expressed Dicer in a subcapsular (arrowhead) location in the sentinel lymph node (SLN) of a patient with metastatic melanoma (MM) compared to the adjacent nodal tissue containing mature lymphocytes (asterisk) that are negative for Dicer. F) In another patient with MM, cancer cells strongly and diffusely expressed Dicer in the SLN, where expanding tumor nodules obliterated the normal lymph node architecture. Under higher magnification, Dicer was localized to the cytoplasm of melanoma cells with a granular quality (inset D and F). Original magnification: A, 200X; B-D, 100X, E, 200X and F, 100X; insets: 400X. (TIF) [file pone.0020494.s001.tif]

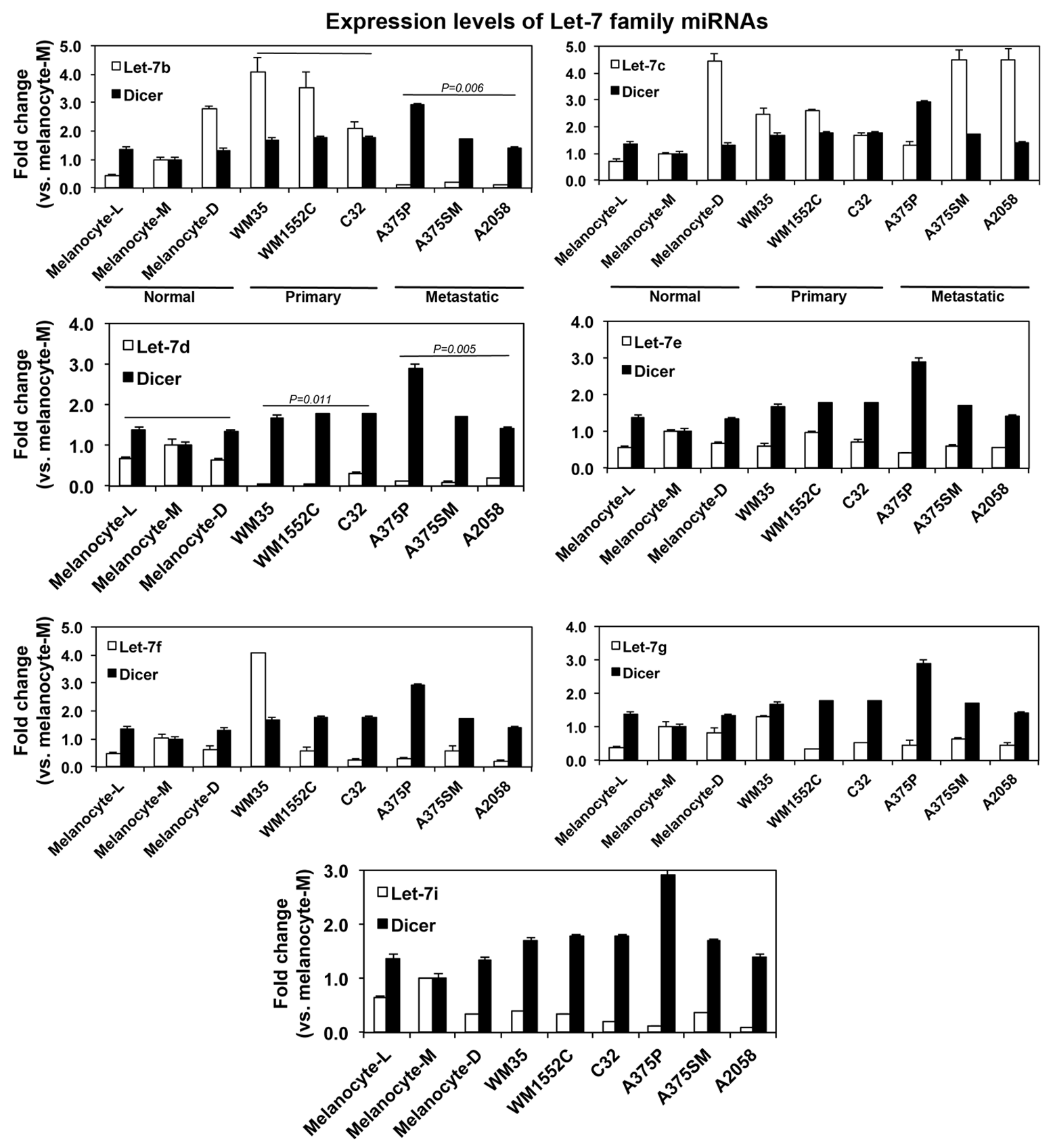

Supplement: Figure S2 — Dicer mRNA expression did not correlate with the expression of any mature miRNA members in the let-7 family in vitro. Using qRT-PCR, the relative expression levels of let-7b, let-7c, let-7d, let-7d, let-7f and let-7g miRNAs and Dicer mRNA were compared to show no significant correlation. However, let-7b expression is significantly down-regulated in all three metastatic compared to three primary melanoma cell lines; whereas let-7d expression is significantly down-regulated in all 6 metastatic and primary melanoma cell lines compared to three melanocytes. All qRT-PCRs were performed in triplicates. Data were normalized to small nuclear RNA RNU6 for let-7 family and GAPDH mRNA for Dicer. (TIF) [file pone.0020494.s002.tif]
