# Supplementary material for: Up-Regulated Dicer Expression in Patients with Cutaneous Melanoma
Source: PLoS One. 2011 Jun 17;6(6):e20494. doi: 10.1371/journal.pone.0020494 (PMC3117784; doi:10.1371/journal.pone.0020494)
Supplement: Table S1 — Pooled analysis performed on enzymes in miRNA biogenesis pathway in publically available gene expression profiling studies (clinical sample size = 139, disease groups = 20). (DOCX) [file pone.0020494.s003.docx]

Table S1. Pooled analysis performed on enzymes in miRNA biogenesis pathway in publically available gene expression profiling studies (clinical sample size=139, disease groups=20).

|  | **Drosha** | | **DGCR8** | | **RAN** | | **XPO5** | |
| --- | --- | --- | --- | --- | --- | --- | --- | --- |
| **Disease Groups** | **P-value** | **Fold change** | **P-value** | **Fold change** | **P-value** | **Fold change** | **P-value** | **Fold change** |
| **Skin biopsy dysplastic nevus low atypia _vs_ common acquired melanocytic nevi** | NS | NS | NS | NS | 3.80E-06 | 2.08 | NS | NS |
| **Skin biopsy dysplastic nevus high atypia _vs_ common acquired melanocytic nevi** | NS | NS | NS | NS | NS | NS | NS | NS |
| **Melanoma in situ _vs_ normal skin** | NS | NS | NS | NS | NS | NS | NS | NS |
| **Primary melanoma _vs_ normal skin** | 0.003 | 2.46 | 0.0138 | -1.21 | 0.0473 | 1.32 | NS | NS |
| **Primary melanoma _vs_ melanoma in situ** | 0.0004 | 3.89 | NS | NS | NS | NS | NS | NS |
| **Melanoma Clark Level I -radial growth _vs_ common acquired melanocytic nevi** | NS | NS | NS | NS | NS | NS | NS | NS |
| **Melanoma Clark Level II -radial growth _vs_ common acquired melanocytic nevi** | NS | NS | NS | NS | NS | NS | NS | NS |
| **Melanoma Clark Level III -vertical growth _vs_ common acquired melanocytic nevi** | NS | NS | NS | NS | 0.0254 | 1.67 | NS | NS |
| **Melanoma Clark Level IV -vertical growth _vs_ common acquired melancytic nevi** | NS | NS | NS | NS | 1.90E-07 | 3.06 | NS | NS |
| **Melanoma Clark Level V -vertical growth _vs_ common acquired melanocytic nevi** | NS | NS | 0.0426 | -1.27 | 4.40E-06 | 2.31 | NS | NS |
| **Metastatic melanoma _vs_ normal skin** | 0.0002 | 2.85 | NS | NS | 0.0006 | 1.76 | 0.0074 | 1.88 |
| **Metastatic melanoma _vs_ melanoma in situ** | 0.0094 | 4.52 | NS | NS | NS | NS | NS | NS |
| **Metastatic melanoma _vs_ primary melanoma** | NS | NS | NS | NS | 0.0266 | 1.33 | NS | NS |
| **Lymph node metastasis of melanoma _vs_ common acquired melanocytic nevi** | NS | NS | 0.0138 | 1.86 | NS | NS | NS | NS |
| **Dermal metastasis of melanoma _vs_ common acquired melanocytic nevi** | NS | NS | NS | NS | 1.50E-06 | 2.22 | NS | NS |
| **Basal cell carcinoma _vs_ normal skin** | 2.10E-09 | 1.69 | 1.00E-05 | 1.55 | 1.90E-03 | 1.63 | 0.0121 | 1.86 |
| **Squamous cell carcinoma of skin _vs_ normal skin** | 0.0007 | 1.47 | NS | NS | 1.50E-05 | 2.55 | 0.0043 | 2.14 |
| **Squamous cell carcinoma of skin _vs_ basal cell carcinom** | NS | NS | 0.0118 | -1.39 | 0.002 | 1.57 | NS | NS |
| **Basal cell carcinoma _vs_ primary melanoma** | NS | NS | 2.30E-08 | 1.88 | NS | NS | NS | NS |
| **Squamous cell carcinoma of skin _vs_ primary melanoma** | NS | NS | NS | NS | 7.00E-05 | 1.93 | NS | NS |
|  |  |  |  |  |  |  |  |  |
|  | **DICER1** | | **GEMIN3** | | **GEMIN4** | | **EIF2C2** | |
|  | **P-value** | **Fold change** | **P-value** | **Fold change** | **P-value** | **Fold change** | **P-value** | **Fold change** |
| **Skin biopsy dysplastic nevus low atypia _vs_ common acquired melanocytic nevi** | 0.0006 | 2.17 | NS | NS | 0.0461 | -1.21 | NS | NS |
| **Skin biopsy dysplastic nevus high atypia _vs_ common acquired melanocytic nevi** | 0.0074 | 1.49 | NS | NS | NS | NS | NS | NS |
| **Melanoma in situ _vs_ normal skin** | NS | NS | 0.0111 | -4.88 | 0.0069 | 1.39 | NS | NS |
| **Primary melanoma _vs_ normal skin** | 0.0082 | -2.36 | NS | NS | 0.011 | -1.65 | 0.012 | 1.4 |
| **Primary melanoma _vs_ melanoma in situ** | 0.0391 | 3.25 | 4.80E-09 | 5.87 | 0.0022 | -2.64 | 2.20E-06 | 1.81 |
| **Melanoma Clark Level I -radial growth _vs_ common acquired melanocytic nevi** | NS | NS | NS | NS | NS | NS | NS | NS |
| **Melanoma Clark Level II -radial growth _vs_ common acquired melanocytic nevi** | NS | NS | NS | NS | NS | NS | NS | NS |
| **Melanoma Clark Level III -vertical growth _vs_ common acquired melanocytic nevi** | NS | NS | NS | NS | NS | NS | NS | NS |
| **Melanoma Clark Level IV -vertical growth _vs_ common acquired melancytic nevi** | NS | NS | NS | NS | 0.0007 | -1.49 | NS | NS |
| **Melanoma Clark Level V -vertical growth _vs_ common acquired melanocytic nevi** | 0.0323 | -2.21 | NS | NS | 0.0014 | -2.24 | NS | NS |
| **Metastatic melanoma _vs_ normal skin** | 0.0045 | -2.42 | 3.40E-06 | 1.52 | 0.0027 | -1.54 | NS | NS |
| **Metastatic melanoma _vs_ melanoma in situ** | 1.70E-06 | -2.04 | 5.00E-27 | 6.42 | 0.0118 | -2.46 | 1.10E-12 | 1.85 |
| **Metastatic melanoma _vs_ primary melanoma** | NS | NS | 3.43E-02 | 1.39 | NS | NS | 0.0338 | 1.59 |
| **Lymph node metastasis of melanoma _vs_ common acquired melanocytic nevi** | 0.0244 | -2.95 | NS | NS | 0.0009 | -2.07 | NS | NS |
| **Dermal metastasis of melanoma _vs_ common acquired melanocytic nevi** | 0.0242 | -1.85 | NS | NS | NS | NS | NS | NS |
| **Basal cell carcinoma _vs_ normal skin** | NS | NS | 1.10E-05 | 1.64 | 0.0039 | 1.38 | NS | NS |
| **Squamous cell carcinoma of skin _vs_ normal skin** | NS | NS | 0.0051 | 1.55 | 0.0005 | 1.55 | 0.0206 | 1.37 |
| **Squamous cell carcinoma of skin _vs_ basal cell carcinom** | NS | NS | NS | NS | NS | NS | NS | NS |
| **Basal cell carcinoma _vs_ primary melanoma** | 0.0055 | 2.54 | 0.0054 | -2.21 | 0.0004 | 2.12 | 0.0022 | 2.11 |
| **Squamous cell carcinoma of skin _vs_ primary melanoma** | 0.0108 | 2.39 | NS | NS | 0.0072 | 1.87 | 0.0086 | 1.87 |

NS-not significant. Fold changes in green are lower and those in red are higher than the disease group to which it was compared. No significant changes were found for either Ago2 or TARBP2P (TRBP) among any of the disease groups.
