# Supplementary material for: Up-Regulated Dicer Expression in Patients with Cutaneous Melanoma
Source: PLoS One. 2011 Jun 17;6(6):e20494. doi: 10.1371/journal.pone.0020494 (PMC3117784; doi:10.1371/journal.pone.0020494)
Supplement: Table S2 — Summary of cell lines, source and type. (DOCX) [file pone.0020494.s004.docx]

Table S2. Summary of cell lines, source and type.

| **Cell line** | **Source** | **Cell type** |
| --- | --- | --- |
| TE 354.T | American Type Culture Collection | BCC |
| Melanocyte-L | ScienCell | Light-color skin primary melanocyte culture |
| Melanocyte-M | ScienCell | Medium-color skin primary melanocyte culture |
| Melanocyte-D | ScienCell | Dark-color skin primary melanocyte culture |
| WM983A | Coriell | PCM^2^, RGP^3^/VGP^4^ |
| WM278 | Coriell | PCM^2^, VGP^4^ |
| WM35 | Wistar institute | PCM^2^, RGP^3^ |
| WM1552C | Wistar institute | PCM^2^, RGP^3^ |
| A2058 | Dr. Stanley N. Cohen | Metastatic melanoma (to LN^5^) |
| A375P | Dr. Stanley N. Cohen | Metastatic melanoma |
| C32 | Dr. Stanley N. Cohen | Amelanotic melanoma |
| A375SM | Dr. Stanley N. Cohen | Metastatic melanoma |

^2^PCM, primary cutaneous melanoma. ^3^RGP, radial growth phase. ^4^VGP, radial growth phase.^5^LN, lymph node.
