# Supplementary material for: Up-Regulated Dicer Expression in Patients with Cutaneous Melanoma
Source: PLoS One. 2011 Jun 17;6(6):e20494. doi: 10.1371/journal.pone.0020494 (PMC3117784; doi:10.1371/journal.pone.0020494)
Supplement: Table S3 — Dicer expression in relation to cutaneous tumors, sex (n = 328) and age (n = 335). (DOCX) [file pone.0020494.s005.docx]

Table S3. Dicer expression in relation to cutaneous tumors, sex (n=328) and age (n=335).

|  | | **Dicer Immunoreactivity** | | | | | | | |  | |
| --- | --- | --- | --- | --- | --- | --- | --- | --- | --- | --- | --- |
|  | |  | **Negative** | | **Low**  **(≤1.5)** | | **High**  **(>1.6)** | | ***P*-Value^1^** | **Mean ± SD** | ***P*-Value** |
|  |  | **Total** | **n** | **%** | **n** | **%** | **n** | **%** |  |  | |
| **Sex** | Male | 187 | 62 | 33.2% | 64 | 34.2% | 61 | 32.6% |  | 1.07 ± 0.97 |  |
|  | Female | 141 | 48 | 34.0% | 48 | 34.0% | 45 | 32.0% | 0.98 | 1.06 ± 0.96 | 0.90^2^ |
| **Age** | ≤ 29 | 36 | 10 | 27.8% | 15 | 41.7% | 11 | 30.5% |  | 1.06 ± 0.78 |  |
|  | 30-39 | 32 | 11 | 34.4% | 12 | 37.5% | 9 | 28.1% |  | 1.03 ± 0.97 |  |
|  | 40-49 | 67 | 19 | 28.4% | 25 | 37.3% | 23 | 34.3% |  | 1.25 ± 0.98 |  |
|  | 50-59 | 68 | 19 | 21.9% | 27 | 39.7% | 22 | 32.4% |  | 1.11 ± 0.97 |  |
|  | ≥ 60 | 132 | 51 | 38.6% | 38 | 28.8% | 43 | 32.6% | 0.72 | 1.07 ± 0.96 | 0.85^3^ |

1. Pearson Chi-Square test for proportions.
2. Mann-Whitney (k=2) non-parametric test for continuous values.
3. Kruskal-Wallis (k=3 or more) non-parametric test for continuous values.
