# Supplementary material for: Up-Regulated Dicer Expression in Patients with Cutaneous Melanoma
Source: PLoS One. 2011 Jun 17;6(6):e20494. doi: 10.1371/journal.pone.0020494 (PMC3117784; doi:10.1371/journal.pone.0020494)
Supplement: Table S4 — Dicer expression in relation to melanoma type and anatomic site (n = 133). (DOCX) [file pone.0020494.s006.docx]

Table S4. Dicer expression in relation to melanoma type and anatomic site (n=133).

|  | | **Dicer Immunoreactivity** | | | | | | | |  | |
| --- | --- | --- | --- | --- | --- | --- | --- | --- | --- | --- | --- |
|  | |  | **Negative** | | **Low**  **(≤1.5)** | | **High**  **(>1.6)** | | ***P*-Value^1^** | **Mean ± SD** | ***P*-Value** |
|  |  | **Total** | **n** | **%** | **n** | **%** | **n** | **%** |  |  | |
| Cutaneous  Melanoma (n=93)^4^ | |  |  |  |  |  |  |  |  |  |  |
|  | Head and Neck | 11 | 1 | 9.1% | 3 | 27.3% | 7 | 63.6% |  | 1.64 ± 0.81 |  |
|  | Upper Extremity | 16 | 1 | 6.3% | 5 | 31.2% | 10 | 62.5% |  | 1.69 ± 0.79 |  |
|  | Trunk | 27 | 7 | 25.9% | 8 | 29.6% | 12 | 44.5% |  | 1.33 ± 1.03 |  |
|  | Lower Extremity | 29 | 6 | 20.7% | 11 | 37.9% | 12 | 41.4% |  | 1.38 ± 1.01 |  |
|  | Genital | 10 | 2 | 20.0% | 5 | 50.0% | 3 | 30.0% | 0.62 | 1.15 ± 0.94 | 0.49^3^ |
| Acrolentiginous Melanoma (n=40) | |  |  |  |  |  |  |  |  |  |  |
|  | Palmar Surface | 12 | 2 | 16.7% | 6 | 50.0% | 4 | 33.3% |  | 1.21 ± 0.89 |  |
|  | Plantar Surface | 28 | 2 | 7.1% | 8 | 28.6% | 18 | 64.3% | 0.19 | 1.79 ± 0.89 | 0.06^2^ |

1. Pearson Chi-Square test for proportions.
2. Mann-Whitney (k=2) non-parametric test for continuous values.
3. Kruskal-Wallis (k=3 or more) non-parametric test for continuous values.
4. Two cases with unknown anatomic site.
